# Long Term Osmotic Mini Pump Treatment with Alpha-MSH Improves Myocardial Function in Zucker Diabetic Fatty Rats

**DOI:** 10.3390/molecules22101702

**Published:** 2017-10-12

**Authors:** Miklos Szokol, Daniel Priksz, Mariann Bombicz, Balazs Varga, Arpad Kovacs, Gabor Aron Fulop, Tamas Csipo, Aniko Posa, Attila Toth, Zoltan Papp, Zoltan Szilvassy, Bela Juhasz

**Affiliations:** 1 Department of Cardiology, Faculty of Medicine, University of Debrecen, H-4032 Debrecen, Hungary; szokol.miklos@med.unideb.hu; 2 Department of Pharmacology and Pharmacotherapy, Faculty of Medicine, University of Debrecen, H-4032 Debrecen, Hungary; priksz.daniel@pharm.unideb.hu (D.P.); bombicz.mariann@pharm.unideb.hu (M.B.); varga.balazs@pharm.unideb.hu (B.V.); szilvassy.zoltan@med.unideb.hu (Z.S.); 3 Division of Clinical Physiology, Faculty of Medicine, University of Debrecen, H-4032 Debrecen, Hungary; kovacs.arpad@med.unideb.hu (A.K.); fulop.gabor@med.unideb.hu (G.A.F.); csipo.tamas@med.unideb.hu (T.C.); atitoth@med.unideb.hu (A.T.); pappz@med.unideb.hu (Z.P.); 4 Department of Physiology, Anatomy and Neuroscience, Faculty of Science and Informatics, University of Szeged, H-6720 Szeged, Hungary; paniko@bio.u-szeged.hu

**Keywords:** alpha-melanocyte-stimulating hormone, echocardiography, isolated working heart, myocyte force, NADPH oxidase

## Abstract

The present investigation evaluates the cardiovascular effects of the anorexigenic mediator alpha-melanocyte stimulating hormone (MSH), in a rat model of type 2 diabetes. Osmotic mini pumps delivering MSH or vehicle, for 6 weeks, were surgically implanted in Zucker Diabetic Fatty (ZDF) rats. Serum parameters, blood pressure, and weight gain were monitored along with oral glucose tolerance (OGTT). Echocardiography was conducted and, following sacrifice, the effects of treatment on ischemia/reperfusion cardiac injury were assessed using the isolated working heart method. Nicotinamide adenine dinucleotide phosphate (NADPH) oxidase activity was measured to evaluate levels of oxidative stress, and force measurements were performed on isolated cardiomyocytes to determine calcium sensitivity, active tension and myofilament co-operation. Vascular status was also evaluated on isolated arterioles using a contractile force measurement setup. The echocardiographic parameters ejection fraction (EF), fractional shortening (FS), isovolumetric relaxation time (IVRT), mitral annular plane systolic excursion (MAPSE), and Tei-index were significantly better in the MSH-treated group compared to ZDF controls. Isolated working heart aortic and coronary flow was increased in treated rats, and higher Hill coefficient indicated better myofilament co-operation in the MSH-treated group. We conclude that MSH improves global heart functions in ZDF rats, but these effects are not related to the vascular status.

## 1. Introduction

Diabetes mellitus type 2 (T2DM) is a long term, life-threatening metabolic disorder characterized by high systemic glucose levels, insulin resistance and damage to many tissues due to dysregulated inflammation [1,2,3]. Unfortunately for those afflicted, this disease persists for a lifetime and progressively causes a wide range of debilitating effects, prominently: retinopathy, nephropathy, myocardial infarction, stroke, and damage to extremities severe enough to require amputation [4,5]. Pharmacotherapy for T2DM includes insulin analogues, biguanids, sulfonylureas, α-glucosidase inhibitors, thiazolidinediones, SGLT-2-inhibitors, GLP-1 analogues, DPP-4-inhibitors, amylin analogues. These agents may partly normalize blood glucose levels and attenuate the severe downstream consequences, allowing management of the disease to some degree in many patients. These therapeutic strategies are nevertheless palliative and generally fail to restore type 2 diabetics to full health. Moreover, epidemiological studies show that incidence of this disorder has dramatically increased to epidemic proportions [6,7,8,9]. Numerous factors have contributed to this phenomenon, principally caused by sedentary lifestyle-related factors such as over-nutrition, lack of exercise and particularly obesity [10,11,12]. Efforts to develop countermeasures to onset and pathogenesis of T2DM, have focused particularly on physiological energy utilization mechanisms. Within this domain, the melanocortin system plays a crucial role regulating whole body energy homeostasis since it functions as a potent anorexigenic influence by controlling appetite resulting in lower food intake [13,14]. A major bioactive molecule in this system, adrenocorticotropic hormone (ACTH) is a peptide derived from pro-opiomelanocortin (POMC). The first 13 amino acids of ACTH (ACTH 1-13) interact with melanocortin receptors MCR1-MCR5 to produce biological effects [15,16,17].

Melanocortins are one of the most important regulators of human food intake behaviour and thus strongly influence development of obesity, with resulting increased risk of T2DM onset or exacerbation. It has also been shown that both peripheral and central injection of melanocortin analogue caused a dramatic alteration in food intake and body weight [18,19,20,21,22]. Furthermore, genetic studies have revealed that mutations in genes coding for components of the melanocortin system correlate with obesity and occurrence of T2DM in both animals and humans [23]. Mechanistic studies reveal that alpha-MSH activates the MC5R signalling pathway via cyclic adenosine monophosphate/protein kinase A (cAMP/PKA) and mitogen-activated protein kinase/extracellular signal-regulated (MAPK/ERK1/2) and significantly decreases the fat content of adipocytes [24,25,26]. In addition to these effects, the beneficial properties of α-MSH have been characterized in many other animal disease models, in which this system exhibits antiapoptotic, anti-inflammatory, antiischemic, antioxidant features [27,28,29,30]. Nevertheless, greater insight into the fact how these molecules may affect cardiomyocyte force during long-term treatment regimens would clearly be beneficial [31,32,33,34]. The present investigation addresses this gap in understanding of cellular processes underlying functions of the melanocortin system. This preclinical study was designed to demonstrate effects of alpha-MSH stimulation sustained over a fairly long time period (6 weeks) on cardio metabolic parameters, diastolic cardiac function, and myofilament co-operation using an osmotic mini pump in the Zucker diabetic fatty rat model. The major objective of the study is to characterize the adaptive/protective role of this peptide on metabolic, cardiac or vascular status of diabetic tissues.

## 2. Results

### 2.1. Weight Gain, Serum Parameters, Blood Pressure and LV Mass/Whole Body Mass Ratio

Initial body weight of animals was 326.70 ± 5.723 g. Weight gain of the animals by treatment groups, serum cholesterol and triglyceride values and results of non-invasive blood pressure measurements are shown in Table 1. No significant differences were found in the abovementioned parameters. On the contrary, significant change was observable between the control and alpha-MSH treated groups in the ratio of left ventricle (LV) mass to whole body mass at the endpoint (Table 1).

### 2.2. Results of OGT Tests

**Figure 1:**
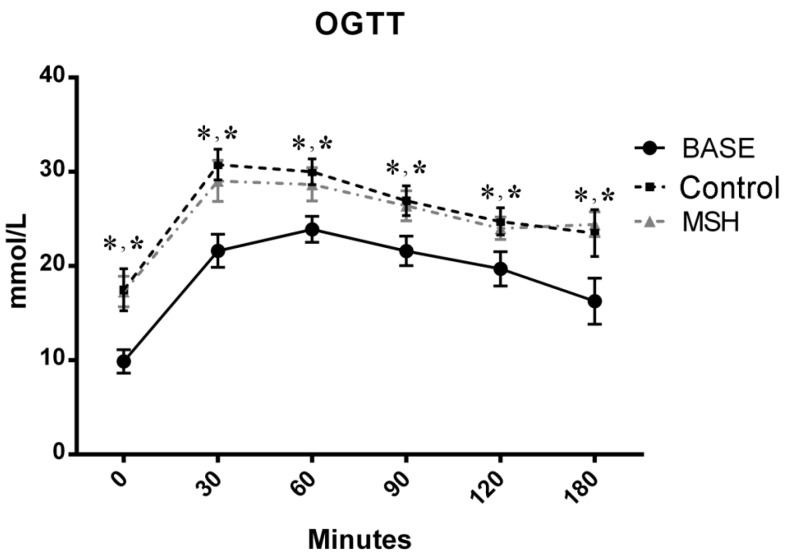
Results of the Oral Glucose Tolerance Test (OGTT) carried out at the start and on the 6th week of the experiment. No significant differences were seen between alpha-MSH-treated and untreated ZDF animal-groups. One-way ANOVA, all data is presented as mean ± SEM. * vs. BASE, *p* < 0.05.

Results of Oral Glucose Tolerance Test (OGTT) are shown on Figure 1. Values of ZDF control and alpha-MSH-treated animals were elevated compared to the baseline (BASE), but no significant changes were observed in MSH group compared to Control at any time points.

### 2.3. Echocardiography

Outcomes of echocardiographic analyses at the start and enpoint are shown in Table 2. Systolic parameters (EF, FS, MAPSE) and diastolic values (E wave velocities, E/e’ ratio, and IVRT) were found to be deteriorated in ZDF Control group compared to baseline (BASE) data (see Table 2). Mild but significant increase in Tei-index (0.491 ± 0.014 vs. 0.305 ± 0.012) shows worsened global heart function. Systolic function of MSH group animals showed a mild improvement in comparison to Control group, demonstrated by fractional shortening (FS), ejection fraction (EF) and mitral annular plane systolic excursion (MAPSE) parameters. FS and EF of alpha-MSH-treated animals were significantly increased in comparison with values of ZDF control animals (FS: 32.33 ± 0.421% vs. 36.83 ± 0.703%; and EF: 66.50 ± 0.067% vs. 72.00 ± 0.774%, respectively). MAPSE values of MSH rats were maintained at the normal range [35,36], however, MAPSE was significantly deteriorated in ZDF control rats (2.268 ± 0.010 mm vs. 1.602 ± 0.045 mm). Diastolic function of the left ventricle was slightly improved in alpha-MSH-treated animals compared to ZDF Controls, demonstrated by a decrease in isovolumic relaxation time (58.00 ± 1.826 ms vs. 43.00 ± 1.125 ms). Diameter of the left atrium was increased in ZDF controls compared to MSH animals showed by left atrium to aortic (LA/Ao) ratios (1.104 ± 0.043 vs. 0.945 ± 0.029). E/A and E/e’ ratios, as well as lateral e’ parameters were found to be unaffected by the treatment. Tei index (Myocardial Performance Index, MPI) was elevated in Control animals when compared to MSH group, showing deteriorated global heart function in Control rats (0.491 ± 0.014 vs. 0.392 ± 0.013). Left ventricle outflow tract (LVOT) parameters were also found to be significantly increased in MSH group compared to ZDF Controls. Alpha-MSH treatment slightly elevates blood flow velocities (V) and pressure gradient (PG) (LVOTV mean: 0.441 ± 0.024 m/s vs. 0.553 ± 0.019 m/s; and LVOT mean PG: 1.095 ± 0.088 mmHg vs. 1.592 ± 0.106 mmHg). Consequently, stroke volume (SV) and cardiac output (CO) were found to be elevated in treated animals (SV: 0.406 ± 0.046 mL vs. 0.581 ± 0.030 mL; and CO: 77.55 ± 7.763 mL/min vs. 112.30 ± 6.110 mL/min, respectively). Heart rate values did not show any difference among groups when measured on anaesthetized animals by echocardiography.

### 2.4. Isolated Working Heart Results

**Figure 2:**
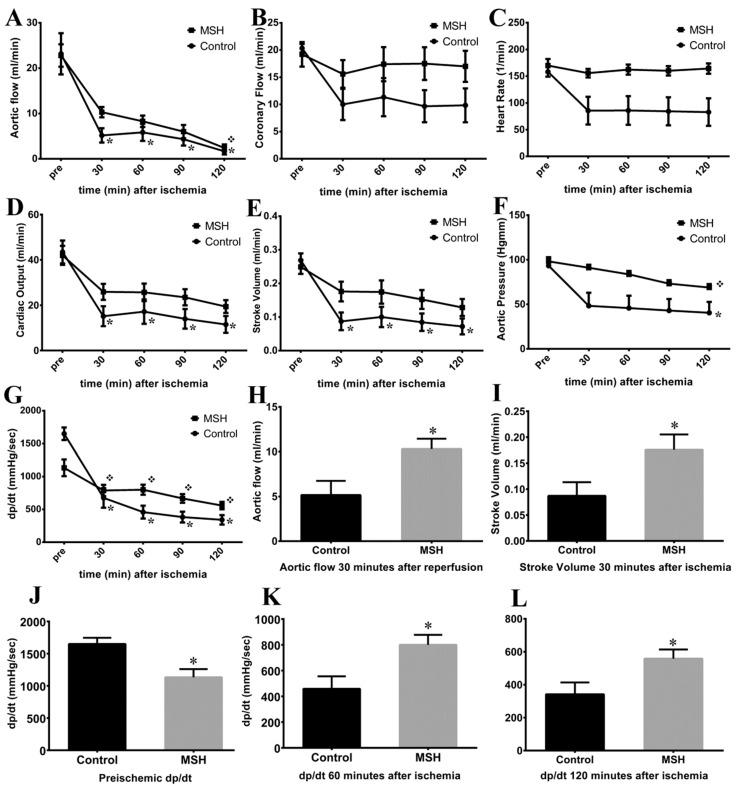
Results of the isolated working heart method. (**A**) aortic flow values; (**B**) coronary flow values; (**C**) heart rate values; (**D**) cardiac output values; (**E**) stroke volume values; (**F**) aortic pressure values; (**G**) dp/dt values; (**H**) aortic flow 30 min after reperfusion; (**I**) stroke volume 30 min after reperfusion; (**J**) pre-ischemic dp/dt values; (**K**) dp/dt 60 min after reperfusion; (**L**) dp/dt 120 min after reperfusion. One-way ANOVA was used (Figure 2A–G), all data is presented as mean ± SEM. * *p* < 0.05 compared to pre-ischemic Control values. ❖ *p* < 0.05 compared to pre-ischemic MSH treated values. * *p* < 0.05 compared to control values at the same time point during isolated working heart experiments (Student’s *t*-test, Figure 2H–L).

The impact of MSH on the ischemic heart was measured by working heart apparatus after 6 weeks following surgery. Six weeks of treatment had no effect on pre-ischemic parameters of contractile function including aortic flow, coronary flow, heart rate, cardiac output, stroke volume and aortic pressure in hearts isolated from either Control or MSH-treated rats (Figure 2).

However, although nearly all parameters showed no differences in pre-ischemic state, in time derivative of developed pressure MSH group featured significantly lower compared to Control group (Figure 2H). Interestingly, in 60 and 120 min of recovery the dp/dt was significantly higher in MSH group than Control parameters in the same time points (Figure 2K,L). Furthermore, 30 min after global ischemia AF and SV were increased in MSH group compared to Control (Figure 2I,J). In addition, at the end of post-ischemic recovery, AF (1.667 ± 0.711; Figure 2A), CO (11.500 ± 3.708; Figure 2D), SV (0.072 ± 0.024; Figure 2E), AoP (40.500 ± 12.230; Figure 2F) and dp/dt (341.700 ± 71.830; Figure 2G) were significantly suppressed in hearts isolated from Control rats compared to their pre-ischemic state (AFpre: 23.170 ± 4.554; COpre: 43.500 ± 5.054; dp/dtpre : 1650 ± 96.120; AoPpre: 93.330 ± 9.482 and SVpre: 0.268 ± 0.021). In contrast, only the post-ischemic AF (2.400 ± 0.778), AoP (68.800 ± 3.952) and dp/dt (558.300 ± 55.630) showed significant reduction in MSH treated group compared to pre-ischemic values (AFpre: 22.800 ± 2.480; dp/dtpre: 1133 ± 127.200; AoPpre: 98.200 ± 2.760). In other words, despite the fact that there were few remarkable differences between the two groups at parallel time points of measurement, in MSH group post-ischemic CO (19.400 ± 2.849) and SV (0.128 ± 0.025) values did not reach statistical significance in reduction compared to their pre-ischemic state (COpre: 42.000 ± 4.195; SVpre: 0.249 ± 0.021). No significance was found in coronary flow and heart rate values, neither between groups, nor between different time points of the same group (Figure 2B,C).

### 2.5. Enhancement of Cardiomyocyte Contractile Performance after Alpha-MSH Treatment

**Figure 3:**
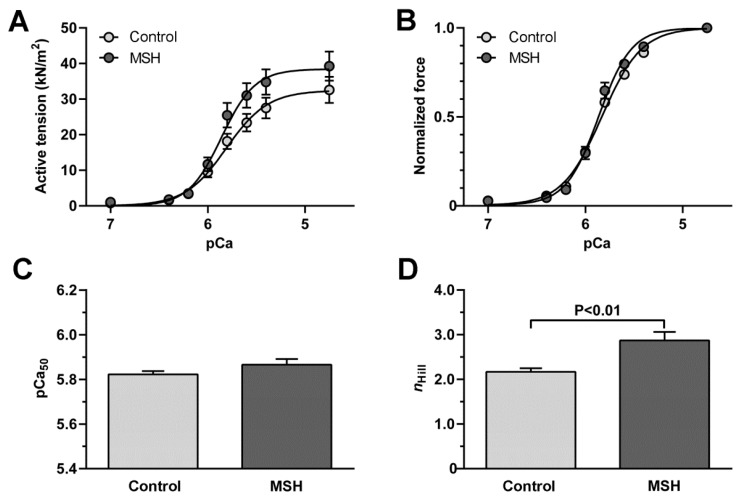
Enhanced contractile performance of left ventricular cardiomyocytes following alpha-MSH treatment. (**A**) Active tension-pCa relationships; (**B**) Normalized force-pCa relationships; (**C**) Half-value of Ca^2+^ sensitivity curves on Panel B indicates myofilament Ca^2+^ sensitivity (pCa_50_); (**D**) Steepness of Ca^2+^ sensitivity curves on Panel B indicates myofilament co-operation as Hill coefficient (*n*_Hill_). Data are given as mean ± SEM, whereas *n* = 12 cardiomyocytes (from 3 to 4 hearts)/groups. P values were calculated by unpaired *t*-test and shown when *p* < 0.05.

Active tension-pCa relationships of LV cardiomyocytes (Figure 3A) apparently showed alpha-MSH-induced changes in cellular mechanical performance of ZDF rats. LV cardiomyocytes from ZDF animals after vs. without alpha-MSH treatment had a trend towards higher active tension (at pCa 5.6: 31.04 ± 3.44 kN/m^2^ vs. 23.38 ± 2.46 kN/m^2^, *p* = 0.08; at pCa 5.8: 25.50 ± 3.43 kN/m^2^ vs. 18.13 ± 2.15 kN/m^2^, *p* = 0.08). Normalized force-pCa relationships of LV cardiomyocytes (Figure 3B) from treated vs. Control ZDF rats showed similar Ca^2+^ sensitivity (pCa_50_: 5.87 ± 0.03 vs. 5.82 ± 0.02; Figure 3C), but significantly higher Hill coefficient (*n*_Hill_: 2.87 ± 0.19 vs. 2.17 ± 0.08; Figure 3D) as indicative for better myofilament co-operation in the alpha-MSH-treated group.

### 2.6. Vascular Status Brain Arteries

**Figure 4:**
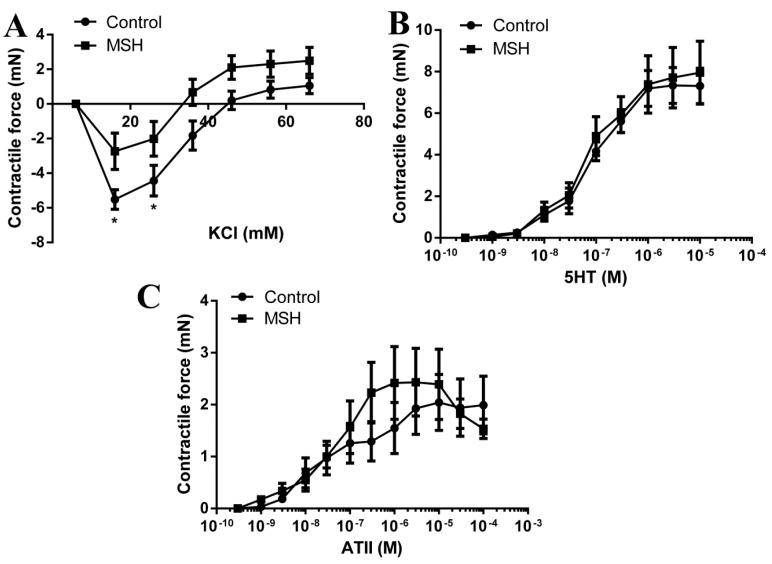
Vascular status of brain arteries—contractile force measurements. (**A**) KCl evoked responses; (**B**) Serotonin (5HT) evoked responses; (**C**) Angiotensin II evoked responses. * *p* < 0.05, all data are presented as mean ± SD.

Significantly higher hyperpolarization induced relaxation in the ZDF Control group compared to alpha-MSH treated group (5.52 ± 0.56 mN in ZDF vs. 2.73 ± 1.05 mN in alpha-MSH treated ZDF *p* < 0.05 at 16 mM KCl) although no difference in the maximal contractile force evoked by 66 mM KCl (1.06 ± 0.466 mN in the Control group vs. 2.49 ± 0.77 mN in the alpha-MSH treated group) was seen during contractile force measurement experiments (Figure 4A).

No difference was found between the 5HT responses of the two groups (7.31 ± 0.85 mN in Control group vs. 7.95 ± 1.51 mN in the alpha-MSH treated group for the maximal 10 μM dose of 5HT; Figure 4B).

There was also no difference in the ATII evoked contractions between the two groups (1.99 ± 0.55 mN in the Control group vs. 1.53 ± 0.188 mN in the alpha-MSH treated group for the maximal 100 μM dose of ATII; Figure 4C).

### 2.7. NADPH Oxidase Activity

**Figure 5:**
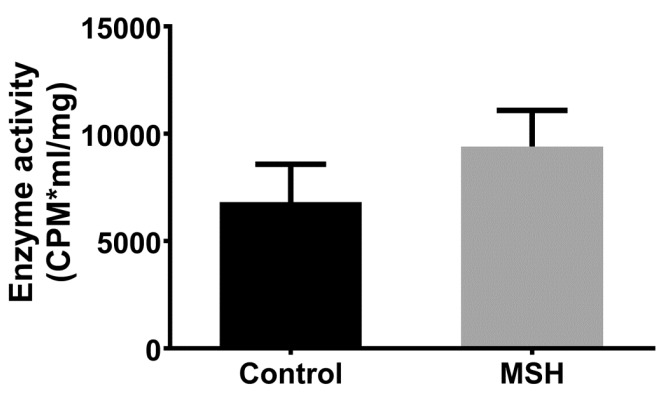
Effects of α-MSH treatment on NADPH stimulated NADPH oxidase activities of left ventricular tissue homogenates. Data are expressed as mean ± SEM (Student’s *t*-test). Samples were measured in duplicates, and the average of the averages are shown. CPM, Count Per Minute; NADPH, Nicotine adenine dinucleotide phosphate; α-MSH, α-Melanocyte-stimulating hormone.

NADPH oxidase activities of left ventricle samples did not differ significantly in the α-MSH treated and untreated Control groups (Figure 5).

## 3. Discussion

Alpha-MSH (α-MSH), a neuropeptide derivative of proopiomelanocortin, is a melanotropin (melanocyte-stimulating hormone) secreted to bind with melanocortin receptors, with resulting downstream physiologic effects, principally food-intake regulation, with resulting weight loss and prevention of obesity—and corollary reduction of diabetes risk [37].

Results of the present investigation included an observation that alpha-MSH-treated animals exhibited lower weight gain than vehicle-treated control rats, however this difference was non-significant for the sample size studied. This outcome notwithstanding, significant differences were noted between alpha-MSH-treated versus control animals in the ratios of left ventricle to whole body mass at the end of the experiment (Table 1). A major implication of this result is that there was greater thickening in the left ventricular walls of control animals versus those treated with the hormone. Since this phenomenon, known as ventricular wall hypertrophy, is associated with increased risk of ischemic heart failure [38,39], treatment with alpha-MSH was for these experiments, assessed as beneficial—with potential for human clinical applications.

At the time of writing, few published descriptions are available that describe correlation between alpha-MSH and blood cholesterol levels. Moreover, results shown here, suggest that alpha-MSH does not directly modulate serum total cholesterol, high density lipoprotein (HDL), or triglycerides. Nevertheless, reports by other investigators demonstrate effects of the hormone on cholesterol-related pathologic processes. For example, investigators in Finland showed that engagement of type 1 and 3 melanocortin receptors—to which alpha-MSH may bind, protects against atherosclerotic plaque-formation, although the mechanism of this process remains obscure[39]. It has also been shown that constitutive cholesterol-dependent endocytosis of the melanocortin-4 receptor (MC4R) is essential for maintenance of receptor responsiveness to alpha-MSH [40]. The above results are intriguing but do not definitively establish existence of direct functional relationships between cholesterol-homeostasis and alpha-MSH activity. It is further worth noting that the ZDF rat model is considered to be non-optimal for evaluation of hypercholesterolemic pathology.

Previous studies have demonstrated the central regulatory role of alpha-MSH in food intake, fat and glucose homeostasis, it reduces appetite and enhances energy expenditure [41,42]. It has also been shown that alpha-MSH is also capable of limiting fat deposition by increasing lipolysis [43,44]. Related studies showed that chronic central and acute peripheral administration of an alpha-MSH analogue, decreased food intake and body weight and limited insulin resistance in a high-fat diet induced obesity mouse study [45]. Investigators conducting the above study further demonstrated a waning of the hormone’s appetite-reducing capacity. This effect was speculated to occur as a consequence of either receptor desensibilisation, or the superimposition of one or two adaptive responses. The present study is the first to evaluate the effect of peripherally administered (via an osmotic pump) alpha-MSH on blood glucose levels in a ZDF rat model of diabetes and obesity. The experimental design parameters used here, were established to enable parallel, comparative studies with previous work, using a variety of models. Data trends observed here, exhibited patterns similar to work by related investigations, albeit without statistical significance (Figure 1).

Echocardiographic outcomes shown in Table 2, reveal the protective effects of long-term alpha-MSH treatment on cardiovascular systolic and diastolic function. This result is relevant to previous work by authors of the present report, in which echocardiographic parameters were monitored using single acute administration of alpha-MSH doses (10, 100 and 250 μg/kg), with resulting significant enhancement of systolic function (EF, FS) [46]. In the present study, the chronic alpha-MSH treatment engendered a similar pattern. Specifically, in animals receiving the hormone, fractional shortening, ejection fraction, stroke volume and cardiac output values were significantly elevated in comparison with control animals (FS: 32.33 ± 0.421% vs. 36.83 ± 0.703%; EF: 66.50 ± 0.067% vs. 72.00 ± 0.774%, SV: 0.41 ± 0.046 mL vs. 0.58 ± 0.030 mL, CO: 77.55 ± 7.763 mL/min vs. 112.30 ± 6.110 mL/min), respectively. Left ventricular outflow tract (LVOT) values are also increased in treated group, demonstrating direct correlation with elevated EF, FS, SV and CO as indicators of systolic function (Table 2). These outcomes should be considered in the context of observations that melanocortin analogues correlate with mild sympathetic activation in the cardiovascular system, including increased heart rate and blood pressure. However, in the present study the systolic and diastolic values measured by in vivo non-invasive tail-cuff blood pressure system, which utilized Volume Pressure Recording (VPR) sensor technology, showed non-significant trends for increase in these values (Table 1). A possible explanation for this result, may be that long acting administration of alpha-MSH, is attenuated in the diseased model—but may nevertheless prove useful future strategies for prevention and therapy of obesity-related syndromes.

Previous investigations have shown that typically alpha-MSH does not affect blood pressure or may only slightly elevate it [34,46]. Moreover, other effects of the hormone on other echocardiographic outcomes shown here, bear consideration. For example, increasing left atrial aortic root ratio (LA/Ao) value indicates means left atrial dilation. Here, alpha-MSH treatment decreased these values, although, both remained in healthy range. MV Deceleration Time (ms), a powerful prognostic marker of LV remodelling and diastolic dysfunction was also considered. Influences that affect MV alters the relationship between early and late filling (E- and A-wave), along with how rapidly flow velocity declines in early diastole (E-wave deceleration time = DT)—and time interval for ventricular filling following relaxation of the ventricle—a value termed: length of the isovolumetric relaxation time (IVRT). α-MSH osmotic pump treatment was observed to significantly improve the diabetes-induced diastolic dysfunction. MV Deceleration Time: 66.67 ± 3.201 vs. 85.50 ± 5.258 ms, and IVRT: 58 ± 1.826 vs. 43 ± 1.125 ms standards were enhanced for hormone-treated animals in comparison with control rats. Other experiments in the present study considered mitral annular plane systolic excursion (MAPSE) also known as left atrioventricular plane displacement (AVPD). Mitral annulus excursion (MAE) or mitral ring displacement is an M-mode-derived echocardiographic marker of LV longitudinal function [47,48,49]. In previous studies reduced MAPSE has been shown to correlate with age, and LV function in patients with myocardial infarction, heart failure and atrial fibrillation [50,51,52,53] and to be more sensitive than conventional echocardiographic markers in detecting abnormalities of LV systolic function at an early stage [7,18]. MAPSE is also known to be prognostic parameter for major cardiac events and mortality in patients with cardiovascular disease [54]. Another measure of heart function considered here, the myocardial performance index (MPI) is an easily performable, recordable and reproducible parameter that may be determined by flow Doppler. In humans, MPI values are independent of arterial pressure, heart rate, ventricular geometry, atrioventricular valve regurgitation, afterload, and preload in patients who are in a supine position. MPI as a single prognostic variable, may be used for assessment of diabetic cardiac dysfunction [55]. The present investigation showed that long-term alpha-MSH treatment significantly improved both parameters in our present study; MAPSE: 1.602 ± 0.045 vs. 2.268 ± 0.010 mm, MPI: 0.491 ± 0.014 vs. 0.392 ± 0.01.

Evaluation of cardiac functions in Langendorff-mounted isolated working hearts shown in Figure 2 revealed significantly increased pre-ischemic pressure change rate (dp/dt) value in MSH group compared to untreated Control group. Also, as shown in Figure 2 ischemic-reperfusion injury-associated decreases in AF, dp/dt, AoP versus pre-ischemic values were observed in both groups by the end of recovery. Despite the fact that nearly all outcome parameters decreased after 120 min of reperfusion, elevated AF and SV were measured at the 30 min recovery time point in the α-MSH-treated group, relative to control animals. Moreover, post-ischemic CO and SV values in animals receiving the hormone did not reach statistical significance in magnitude of suppression, versus their pre-ischemic state. These findings are consistent with previous studies by the authors, in which alpha-MSH treatment significantly inhibited the extent of ischemia/reperfusion-induced infarct zones, increased the magnitude of CO and SV, therefore provides additional evidence of the range of cardio-protective effects mediated by the hormone [46]. These outcomes notwithstanding, little investigative work has addressed this issue at the time of the present writing and awaits further exploration. Significantly, related studies have demonstrated that melanocyte-stimulating hormone administered before ischemia, increases the effectiveness of post-ischemic recovery of function [46,56,57]. Further, recent work by authors of the present report and others, demonstrate that melanocortins protect against tissue damage in response to prolonged myocardial ischemia/reperfusion via activation of pro-survival HO-1 protein, JAK/ERK/STAT signalling and decreased expression of the pro-inflammatory mediator TNF-α and pro-inflammatory/pro-apoptotic factor pJNK and also by vagus nerve-mediated cholinergic anti-inflammatory pathway [46,57,58]. Analysis of results of cardiomyocyte contractile performance data, showed improved actin-myosin co-operation and strong trends toward a higher active tension—suggesting improved cardiomyocyte mechanical performance that may contribute to the beneficial effects of alpha-MSH on global cardiac contractility (Figure 5). In contrast, it appears that MSH had no effect on Ca^2+^ sensitivity of the contractile apparatus. Moreover, it was also noted that the effects of hormone treatment on cerebral vascular smooth muscle, produced no deleterious effects. Specifically, the treatment did not alter values for KCl, serotonin, or responses evoked by angiotensin II (Figure 4)—indicating an apparent lack of vascular response to α-MSH stimulation (Figure 3).

In accordance with previous studies, one of the major hypotheses which may account for the protective effect of the hormone was its immunomodulatory, anti-inflammatory and antioxidant properties [59,60]. These phenomena were a major rationale for aspects of experimental design used in the present study, involving assessment of NADPH oxidase enzyme activity, outcomes of which are shown in Figure 4. Nevertheless, a limitation of the current study is that we only investigated the hormone’s antioxidant capacity by measuring NADPH oxidase activity. Other possible mechanisms are under intensive research to clarify beneficial effects of the hormone.

## 4. Materials and Methods

### 4.1. Animal Model and Chemicals

Male Zucker Diabetic Fatty (ZDF-*Lepr*^fa^) rats weighing 300–350 g (at age of 10 weeks, *n* = 12) were provided by Charles River International Ltd. (Wilmington, MA, USA). Animals were housed under a 12–12 h light-dark cycle and were kept on Purina 5008 special chow (LabDiet, St. Louis, MO, USA) and tap water, as recommended by the distributor. All experimental protocols were approved by the local Ethics Committee of University of Debrecen and the animals received humane care in accordance with the “Principles of Laboratory Animal Care” by EU Directive 2010/63/EU. A 4-week adaptation period was provided to animals before the start point of the study. Alpha-MSH and all other chemicals used for buffer solutions were obtained from Sigma-Aldrich Co. (Budapest, Hungary).

### 4.2. Study Design

After recording baseline data (*n* = 12), including weight, OGT test and echocardiographic recordings, rats were randomly divided into 2 subgroups as follows: untreated animals (Control; *n* = 6) receiving vehicle (saline solution), and animals (MSH; *n* = 6) receiving alpha-MSH solution, both subcutaneously administered by mini-osmotic pumps (as detailed in Section 4.3), for 6 weeks. A limitation of this current report is that we do not present data about lean control animals. Alpha-MSH concentration was adjusted to 4.8 mg/mL, and the pump delivered 0.15 μL/h (0.72 μg alpha-MSH in each hour). After six weeks, study was terminated, endpoint data (weight, OGTT, echocardiography, serum parameters and blood pressure) was recorded, and rats were sacrificed by thoracotomy under deep anaesthesia by ketamine/xylazine combination (CALYPSOL^®^ (ketamine), Richter Gedeon Plc., Budapest, Hungary; NERFASIN^®^ (xylazine) Le Vet. Pharma BV, Oudewater, The Netherlands). Hearts and basilar arteries were excised. Isolated working heart method was carried out, and cardiac tissue samples were rapidly frozen for NADPH oxidase activity and for myocyte force measurements. Basilar arteries were subjected to contractile force studies.

### 4.3. Osmotic Pump Implant Surgery

Alzet^®^ osmotic mini-pumps (Durect Corp., Cupertino, CA, USA) were surgically implanted into a 1 cm opening in the nape skin of ZDF rats under ketamine/xylazine (100/5 mg/kg) deep anaesthesia. Before implantation, the pumps were primed by injecting them with alpha-MSH solution (200 μL solution at the concentration of 4.8 mg/mL) and placing them into 37 °C physiological SAL for a minimum of 4 h [61]. Following insertion of the mini-pumps, the skin was closed with surgical sutures, and the wound was disinfected with Betadine^®^ solution (Egis Pharmaceuticals PLC, Budapest, Hungary). Surgery time was 10–15 min, and the rats received postoperative care. The pump provided continuous administration of 0.15 μL content per hour. The mini-pumps of the control group were filled with physiological SAL and were implanted with the same methods.

### 4.4. Oral Glucose Tolerance Test (OGTT)

OGTT for ZDF rats was performed according to the standard method. Briefly, all animals were selected for OGT test at baseline and at the endpoint, after starving at water for 12 h. 3 g/kg glucose was administered in 1 g/mL solution for each animal via gavage technique [62]. The serum blood glucose levels were obtained by pricking the tail vein and using glucometer (Accu-chek, Roche Diagnostic, Indianapolis, IN, USA) at time 0 (baseline), 30, 60, 90, 120 and 180 min.

### 4.5. Blood Pressure Measurement of Conscious Rats

At the endpoint, systolic blood pressure (SBP) and diastolic blood pressure (DBP) were measured by a non-invasive tail-cuff blood pressure system utilizes Volume Pressure Recording (VPR) sensor technology, (CODA™ Surgical Monitor, Kent Scientific Corp., Torrington, CT, USA). Measurements were obtained in conscious rats restrained in a thermal plastic chamber as described elsewhere [63]. Five consecutive recordings were evaluated in each animal to ensure results.

### 4.6. Analysis of Serum Parameters

At the endpoint of the treatment, after 14-h fasting, rat blood samples were collected from the tail vein in EDTA-K2 evacuated tubes (BD Vacutainer, Franklin Lakes, NJ, USA). The samples were collected and processed aseptically to minimize haemolytic activity. Serum glucose, cholesterol and triglyceride were detected by Department of Laboratory Medicine at the University of Debrecen.

### 4.7. Echocardiographic Studies

Echocardiographic imaging was carried out according the protocol detailed previously by the authors [35,36]. Briefly, measurements were performed under intramuscular anaesthesia (ketamine 50 mg/kg, xylazine 5 mg/kg) at the baseline and at the endpoint of the study. The chest hair was shaved and the animals were positioned in a dorsal position. Data acquisition was performed using a Vivid E9 sonograph (GE Healthcare, New York, NY, USA), with an i13L linear array probe at 14 MHz with high temporal and spatial resolution [64,65,66,67]. Complete 2-dimensional, M-mode (at papillary muscle levels), Doppler (PW), and tissue Doppler (TVI) echocardiograms were acquired and digitally stored for further analysis as recommended by the American Society of Echocardiography. ECG was continously monitored during echocardiographic examinations in all cases. Obtained M-mode, Doppler and tissue Doppler parameters included the followings: aortic root diameter (Ao), left atrial diameter (LA), interventricular septum thickness in diastole and systole (IVSs, IVSd), left ventricular internal diameter at end-diastole (LVIDd) and end-systole (LVIDs), left ventricular ejection fraction (EF), fractional shortening (FS), stroke volume (SV, calculated), cardiac output (CO, calculated), heart rate (HR), mitral annular peak systolic excursion (MAPSE), left ventricular peak E and peak A waves (mitral early and late filling velocities), the E to A ratio (E/A), ejection time (ET), isovolumic contraction- and relaxation time (IVCT and IVRT), myocardial performance index (MPI or Tei-index), lateral e’ and a’ wave velocities, lateral e’/a’ ratio, E/e’ ratio. Left ventricular outflow tract (LVOT) maximal and mean velocity and pressure parameters: LVOT Vmax, Vmean, LVOT maxPG and meanPG. LV mass was calculated by echocardiography, using the following formula: LV Mass (g) = 0.8{1.04[([LVIDd + IVSd + PWd]^3^ − LVIDd^3^)]} + 0.6; where LVIDd is the diameter of the left ventricle in end-diastole; IVSd is thickness of the interventricular septum in diastole; PWd is the thickness of the posterior wall in diastole. Acquired images were analysed using the EchoPAC PC software (GE Healthcare, New York, NY, USA) by a blinded reader.

### 4.8. Isolated Working Heart Preparation

All animals (*n* = 12) were anesthetized with ketamine/xylazine (100/5 mg/kg, i.m.). A bolus of heparin was administered (1000 U/kg, intravenously) 20 min before the thoracotomy, to avoid the thrombosis. Thoracotomies were performed on each animal; the hearts were excised and placed into ice-cold modified Krebs-Henseleit perfusion buffer (mKH buffer). The aortas were then cannulated, and hearts were perfused according to Langendorff method for a 5-min washout period at a constant perfusion pressure equivalent to 100 cm of water (10 kPa). The perfusate was filtered (5 μm pore size) in-line and had the following composition: NaCl, 118 mmol/L; NaHCO_3_, 25 mmol/L; KCl, 4.8 mmol/L; CaCl_2_, 1.8 mmol/L; Mg_2_SO_4_, 1.2 mmol/L; KH_2_PO_4_, 1.2 mmol/L; and glucose, 11 mmol/L. A dual-headed peristaltic pump controlled the rate of perfusion of mKH buffer. Following retrograde perfusion, working-mode perfusion was performed cardiac function was assessed as described previously [68]. Heart rate (HR), aortic pressure (AoP), left ventricular developed pressure (LVDP) were recorded using a PowerLab (ADInstruments) aortic flow (AF) and coronary flow (CF) were measured by using flowmeter, stroke volume (SV) and cardiac output (CO) were further derived from these measures [69,70,71]. Hearts were then maintained to 30 min of global ischemia. After ischemia, hearts were perfused for 15 min in Langendorff mode and then converted to working heart mode for 105 min. The above-mentioned parameters were measured and recorded during the reperfusion at the 30, 60, 90 and 120 min. Immediately after 120 min reperfusion small myocardial biopsies from LV heart tissue were removed and frozen for further analysis [72].

### 4.9. Force Measurements of Isolated Cardiomyocytes

Contractile function of skinned left ventricular (LV) cardiomyocytes (*n* = 12 per group) from isolated working hearts (*n* = 3–4) after the protocol of ischemia/reperfusion (I/R) was measured as it described previously [73]. Briefly, deep-frozen (−80 °C) tissue samples were mechanically disrupted and demembranated by 0.5% Triton X-100 detergent for 5 min in isolating solution (MgCl_2_: 1.0 mM; KCl: 100.0 mM; EGTA: 2.0 mM; ATP: 4.0 mM; imidazole: 10.0 mM; pH 7.0) at 4 °C. Each subjected cell was attached with silicone adhesive at one end to a stainless steel insect needle connecting to a high-speed length controller (Aurora Scientific Inc., Aurora, ON, Canada), while at the other end to a stainless steel insect needle connecting to a sensitive force transducer (SensoNor AS, Horten, Norway) at 15 °C. Subsequent cardiomyocyte isometric force generation was recorded at sarcomere length of 2.3 μm and analysed by LabVIEW software (National Instruments Corp., Austin, TX, USA). Ca^2+^-dependent force production of a single cardiac cell was induced by transferring the preparation from relaxing (BES: 10.0 mM; KCl: 37.11 mM; MgCl_2_: 6.41 mM; EGTA: 7.0 mM; ATP: 6.94 mM; creatine phosphate: 15.0 mM; pH7.2) to activating solutions (same composition as relaxing solution aside from containing Ca^2+^-EGTA instead of EGTA). Ca^2+^ concentrations were indicated as −log10[Ca^2+^] units (pCa). Protease inhibitors were added to all solutions freshly: phenylmethylsulfonyl fluoride: 0.5 mM; leupeptin: 40 μM and E-64: 10 μM. All chemicals were purchased from Sigma-Aldrich Corp. (St. Louis, MO, USA). Cardiomyocyte Ca^2+^-activated force generation was registered by using maximal activating solution (pCa 4.75) and activating solutions with different pCa (5.4–7.0). Submaximal forces were normalized to maximal force and fitted to a modified Hill equation in Origin 6.0 analysis program (OriginLab Corp., Northampton, MA, USA). The pCa value for the half-maximal contraction indicated by pCa50 defines per se the Ca^2+^ sensitivity of force generation of the contractile machinery, while the steepness of the Ca^2+^ sensitivity curve expressed as a coefficient (nHill) reflects the co-operation between the myofilaments. Original active forces were normalized to myocyte cross-sectional area indicating active tension (expressed in kN/m^2^). Statistical analysis was performed by GraphPad Prism 5.02 software (GraphPad Software Inc., La Jolla, CA, USA). Data were compared with unpaired Student’s *t*-test.

### 4.10. Arterial Contractile Force Measurement

During the isolated heart experiments, after thoracotomies performed under ketamine/xylazine anaesthesia, the brain was removed and placed into a silicone containing petri dish, filled with 0–4 °C Krebs solution (composition in mmol: 110 NaCl, 5.0 KCl, 1.0 MgSO_4_, 1.0 KH_2_PO_4_, 5.0 glucose and 24.0 NaHCO_3_, obtained from Sigma-Aldrich, St. Louis, MO, USA). The solution was equilibrated previously with a gaseous mixture of 5% CO_2_, 10% O_2_ and 85% N_2_ at pH 7.4. Basilar arteries were isolated with microsurgical tools (Fine Science Tools GmbH, Heidelberg, Germany). The arteries were equally cut into 4 mm long rings, which were then mounted in an isometric contraction measurement system (DMT-510, Danish Myotechnology, Aarhus, Denmark). The Ca^2+^-free Krebs solution was changed to Ca^2+^-containing one (composition in mM: 110 NaCl, 2.5 CaCl_2,_ 5.0 KCl, 1.0 MgSO_4_, 1.0 KH_2_PO_4_, 5.0 glucose and 24.0 NaHCO_3_, obtained from Sigma-Aldrich, St. Louis, MO, USA). Before every experiment, a normalization protocol was performed, by stretching the preparations with 1.5 mN force, which was increasing evenly every 15 s until the calculated intraluminar pressure reached 13.4 kPa. The experiments were then performed at this stretch level. Contractile responses for KCl (6–66 mM), serotonine (1 nM–10 μM) and angiotensin II (1 nM–100 μM) have been recorded.

### 4.11. NADPH Oxidase Activity Measurement

NADPH oxidase-derived superoxide production was measured using lucigenin-enhanced chemiluminescence, as described previously [74,75]. Left ventricular (LV) samples were snap frozen immediately after termination of ex vivo perfusion experiments and were stored on −70 °C until subsequent analyses. Approximately 100 mg (wet tissue) of LV heart samples (*n* = 4 from both α-MSH treated and untreated) were homogenized (Pro200 homogenizer, Proscientific, Oxford, CT, USA) in 10 volumes of ice cold 1× Ca^2+^-free Dulbecco’s Phosphate Bufferred Saline (Gibco^®^, Thermo Fisher, Waltham, MA, USA) containing 40 μM leupeptin, and 5 μM E64 protease inhibitors. After centrifuging, the pellet was discarded and Bicinchoninic Acid Assay was used to determine protein concentration in the supernatant using bovine serum albumin (BSA) as a standard (all chemicals from Sigma-Aldrich, St. Louis, MO, USA). Protein concentrations were ~20 mg/mL. The reaction mixture for the measurement of NADPH oxidase enzyme activity contained 50 μL heart homogenate in a buffer of Krebs- (4-(2-hydroxy-ethyl)-1-piperazineethanesulfonic acid (HEPES) (110 mM NaCl, 5 mM KCl, 1 mM MgSO_4_, 1 mM KH_2_PO_4_, 5 mM glucose, 24 mM NaHCO_3_, 20 mM HEPES, 50 μM lucigenin). The scintillation tubes went through dark adaptation, and basal luminescence of samples were recorded via PerkinElmer Tricarb 2800 tr liquid scintillation counter (PerkinElmer, Waltham, MA, USA). Enzymatic reaction was stimulated by adding 100 μM NADPH to the reaction mixture. Luminescence was recorded directly after for 2 min. Differences between basal and stimulated luminescence were calculated and values were normalized to protein concentration.

### 4.12. Statistical Analyses

All data are presented as the average magnitudes of each outcome in a group ± standard error of the mean (SEM). The D’Agostino & Pearson omnibus normality test was used to estimate Gaussian distribution. Statistical analysis was performed using one-way analysis of variance (ANOVA) with Bonferroni post-testing (when normality test was passed) or by Kruskal-Wallis test with Dunn’s post-testing (if the normality test was not passed). Student’s *t*-test was used to determine of significance in the case of comparing significance of two groups. Statistical analyses were conducted using GraphPad Prism software for Windows, version 5.01 (GraphPad Software Inc., La Jolla, CA, USA). Probability values (*p*) less than 0.05 were considered statistically significant.

## 5. Conclusions

Long term α-MSH treatment using a mini osmotic pump has significant potential for prevention of and therapy for diabetes-induced systolic or diastolic dysfunction which is not associated with Ca^2+ ^sensitivity and/or vascular status. The study further suggest that ischemia/reperfusion induced cardiac damages are also attenuated but through mechanisms that appear to occur independently from the hormone’s antioxidant features.

## Figures and Tables

**Table 1:** Weight, weight gain, left ventricle (LV) mass and body weight ratio (measured by echocardiography), serum parameters and blood pressure results of untreated control (*n* = 6) and alpha-MSH-treated (*n* = 6) Zucker Diabetic Fatty (ZDF) rats. No significant changes were found in weight gain, plasma cholesterol and triglyceride and blood pressure values among groups. Even though decreased LV mass to body weight ratios were measured in the melanocyte stimulating hormone (MSH) group at the endpoint when compared to Control. * vs. Control, *p* < 0.05, Student’s *t*-test.

Parameter	Control	MSH
Endpoint weight (g)	358.7 ± 7.154	391.8 ± 17.00
Weight gain (%)	20.23 ± 2.444	17.65 ± 2.225
Baseline LV mass/Baseline bodyweight (%)	0.337 ± 0.011	0.321 ± 0.017
Endpoint LV mass/Endpoint bodyweight (%)	0.320 ± 0.011	0.2770 ± 0.010 *
Total cholesterol (mmol/L)	3.445 ± 0.210	3.295 ± 0.074
HDL (mmol/L)	2.082 ± 0.194	1.768 ± 0.092
Triglyceride (mmol/L)	3.487 ± 0.318	3.068 ± 0.344
Systolic BP (mmHg)	134.00 ± 4.073	142.2 ± 1.900
Diastolic BP (mmHg)	96.25 ± 3.484	105.5 ± 4.400

**Table 2:** Echocardiographic parameters of untreated control and alpha-MSH-treated ZDF rats at the baseline and at the endpoint of the study. Ejection fraction (EF), fractional shortening (FS), stroke volume (SV), cardiac output (CO) and mitral plane systolic excursion (MAPSE) were elevated in treated group. Isovolumic relaxation time (IVRT) and isovolumic contraction time (IVCT) were lengthened in ZDF animals, but shortened in alpha-MSH-treated group. Myocardial Performance Index (MPI or Tei-index) and left atrium to aortic ratio (LA/Ao) were also improved after the treatment. One-way ANOVA was used to estimate statistical differences. ^❖^ vs. BASE, *p* < 0.05; * vs. Control, *p* < 0.05.

Parameter	BASE	Control	MSH
LA/Ao ratio	1.133 ± 0.039	1.104 ± 0.043	0.945 ± 0.029 *
LV Ejection Fraction (%)	73.17 ± 1.973	66.50 ± 0.067 ^❖^	72.00 ± 0.774 *
LV Fractional Shortening (%)	37.50 ± 1.500	32.33 ± 0.421 ^❖^	36.83 ± 0.703 *
IVSd (mm)	1.845 ± 0.164	1.550 ± 0.044	1.613 ± 0.099
LVIDd (mm)	7.038 ± 0.048	7.747 ± 0.328	7.688 ± 0.248
IVSs (mm)	2.603 ± 0.075	2.143 ± 0.093 ^❖^	2.097 ± 0.103
LVIDs (mm)	4.385 ± 0.103	5.232 ± 0.226	4.858 ± 0.117
Stroke volume (mL)	0.428 ± 0.037	0.406 ± 0.046	0.581 ± 0.030 *
Cardiac Output (mL/min)	99.89 ± 8.236	77.55 ± 7.763	112.30 ± 6.110 *
HR (bpm)	235.8 ± 8.462	192.7 ± 4.185 ^❖^	193.3 ± 6.259 ^❖^
LVOT maxPG (mmHg)	3.173 ± 0.217	2.698 ± 0.254	3.765 ± 0.284 *
LVOT meanPG (mmHg)	1.178 ± 0.138	1.095 ± 0.088	1.592 ± 0.106 *
LVOT Vmax (m/s)	0.887 ± 0.029	0.818 ± 0.038	0.965 ± 0.036 *
LVOT Vmean (m/s)	0.447 ± 0.032	0.441 ± 0.024	0.553 ± 0.019 *
Lateral e’ (mm/s)	39.50 ± 1.803	26.83 ± 1.939 ^❖^	28.33 ± 0.614
MV E velocity (m/s)	0.887 ± 0.025	0.743 ± 0.014 ^❖^	0.710 ± 0.027
MV A velocity (m/s)	0.477 ± 0.027	0.438 ± 0.022	0.403 ± 0.018
MV E/A ratio	1.885 ± 0.109	1.802 ± 0.048	1.770 ± 0.101
MV Deceleration Time (ms)	55.67 ± 3.333	66.67 ± 3.201	85.50 ± 5.258 *
E/e’ ratio	22.59 ± 0.832	28.47 ± 2.220 ^❖^	25.11 ± 1.070
MAPSE (mm)	2.167 ± 0.061	1.602 ± 0.045 ^❖^	2.268 ± 0.010 *
Ejection Time (ms)	100.7 ± 2.459	83.17 ± 2.926 ^❖^	93.17 ± 3.877
IVCT (ms)	18.50 ± 1.708	23.50 ± 1.727	17.83 ± 0.703 *
IVRT (ms)	25.00 ± 1.390	58.00 ± 1.826 ^❖^	43.00 ± 1.125 *
MPI (Tei-index)	0.305 ± 0.012	0.491 ± 0.014 ^❖^	0.392 ± 0.013 *
